# Effects of short-term dietary nitrate supplementation on exercise and coronary blood flow responses in patients with peripheral artery disease

**DOI:** 10.3389/fnut.2024.1398108

**Published:** 2024-07-03

**Authors:** Danielle Jin-Kwang Kim, Zhaohui Gao, Jonathan C. Luck, Kristen Brandt, Amanda J. Miller, Daniel Kim-Shapiro, Swati Basu, Urs Leuenberger, Andrew W. Gardner, Matthew D. Muller, David N. Proctor

**Affiliations:** ^1^Penn State Heart and Vascular Institute, Penn State College of Medicine, Hershey, PA, United States; ^2^Department of Physics, Wake Forest University, Winston-Salem, NC, United States; ^3^Department of Medicine, University of Oklahoma Health Sciences Center, Oklahoma City, OK, United States; ^4^School of Medicine, Case Western Reserve University, Cleveland, OH, United States; ^5^Noll Laboratory, Department of Kinesiology, Penn State University, University Park, PA, United States

**Keywords:** dietary nitrate supplementation, coronary blood flow velocity, treadmill exercise, isometric handgrip exercise, plantar flexion, peripheral artery disease, plasma nitrite

## Abstract

**Background:**

Peripheral arterial disease (PAD) is a prevalent vascular disorder characterized by atherosclerotic occlusion of peripheral arteries, resulting in reduced blood flow to the lower extremities and poor walking ability. Older patients with PAD are also at a markedly increased risk of cardiovascular events, including myocardial infarction. Recent evidence indicates that inorganic nitrate supplementation, which is abundant in certain vegetables, augments nitric oxide (NO) bioavailability and may have beneficial effects on walking, blood pressure, and vascular function in patients with PAD.

**Objective:**

We sought to determine if short-term nitrate supplementation (via beetroot juice) improves peak treadmill time and coronary hyperemic responses to plantar flexion exercise relative to placebo (nitrate-depleted juice) in older patients with PAD. The primary endpoints were peak treadmill time and the peak coronary hyperemic response to plantar flexion exercise.

**Methods:**

Eleven PAD patients (52–80 yr.; 9 men/2 women; Fontaine stage II) were randomized (double-blind) to either nitrate-rich (Beet-IT, 0.3 g inorganic nitrate twice/day; BR_nitrate_) or nitrate-depleted (Beet-IT, 0.04 g inorganic nitrate twice/day, BR_placebo_) beetroot juice for 4 to 6 days, followed by a washout of 7 to 14 days before crossing over to the other treatment. Patients completed graded plantar flexion exercise with their most symptomatic leg to fatigue, followed by isometric handgrip until volitional fatigue at 40% of maximum on day 4 of supplementation, and a treadmill test to peak exertion 1–2 days later while continuing supplementation. Hemodynamics and exercise tolerance, and coronary blood flow velocity (CBV) responses were measured.

**Results:**

Although peak walking time and claudication onset time during treadmill exercise did not differ significantly between BR_placebo_ and BR_nitrate_, the diastolic blood pressure response at the peak treadmill walking stage was significantly lower in the BR_nitrate_ condition. Increases in CBV from baseline to peak plantar flexion exercise after BR_placebo_ and BR_nitrate_ showed a trend for a greater increase in CBV at the peak workload of plantar flexion with BR_nitrate_ (*p* = 0.06; Cohen’s *d* = 0.56).

**Conclusion:**

Overall, these preliminary findings suggest that inorganic nitrate supplementation in PAD patients is safe, well-tolerated, and may improve the coronary hyperemic and blood pressure responses when their calf muscles are most predisposed to ischemia.

**Clinical trial registration:**https://clinicaltrials.gov/, identifier NCT02553733.

## 1 Introduction

Peripheral artery disease (PAD) is characterized by atherosclerotic lesions in conduit arteries that limit blood flow and oxygen delivery to the leg muscles, particularly during walking and other daily functional demands (1). A common symptom of PAD is intermittent claudication, defined as lower extremity pain that is induced by walking and is only relieved by rest (2). These patients thus have a high perception of disability, greatly restricted daily activities, and reduced quality of life (3–5). PAD affects an estimated 202 million people worldwide and 8–12 million adults in the US (6, 7) and is projected to increase until 2030 with rising metabolic disorders and an aging world (8).

The pathophysiology and burden of PAD extends beyond the lower limb circulation and poor walking tolerance, as these patients also have a high risk of coronary events and cardiac death (9). PAD is, in fact, a diffuse disease affecting both the lower and upper extremities (10) and the heart (9). Previous research on these patients has shown impaired exercise-induced hyperemic responses in each of these vascular beds (11). This includes recent studies in our lab showing attenuated coronary hyperemic responses in PAD patients compared to healthy controls during isolated calf and forearm exercise (12). Reduced endothelial-derived nitric oxide (NO) production and bioavailability has been implicated in each of these vascular impairments (13, 14). It is also well established that patients with PAD have exaggerated blood pressure responses to exercise, although this appears to occur most frequently during dynamic exercise activities involving the lower extremities (1, 15–17).

Non-surgical therapies for patients with PAD have been focused on improving maximal walking distance and reducing cardiac disease risk (18, 19). However, the therapeutic options to achieve these outcomes in this population remain extremely limited. Supervised exercise (treadmill walking) training helps these patients walk further, reduce cardiovascular risk, and remain functionally independent (supported by Level 1a evidence) (20). Unfortunately, adherence to exercise training programs is low due in part to the discomfort experienced by these patients when they walk (20). Several medications have been evaluated for use in patients with claudication symptoms, but efficacy has only been reported for cilostazol and anti-platelet agents, and these can have negative side effects (21). Novel, non-pharmacological interventions that enhance the capacity to walk while also improving blood pressure responses and limiting myocardial ischemia during exertion in these patients are clearly needed.

Inorganic nitrate is a precursor for NO via the nitrate-nitrite-NO pathway, the latter step of which is greatly enhanced during ischemic conditions (22–24). Dietary nitrate supplementation has thus been of particular interest as a potential therapeutic for patients with PAD, who exhibit widespread impairment in endothelium-dependent NO production and tissue ischemia. However, published reports employing dietary nitrate supplementation in PAD are still relatively few in number, limited in their study design (all but one employed a parallel study design) and blinding procedures (several were single- or unblinded), and variable with respect to nitrate’s impact on exercise tolerance and blood pressure/vascular outcomes (25–28). In addition, no studies have measured coronary or myocardial responses following dietary nitrate supplementation in PAD. The primary aims of the present study were, accordingly, to determine if short-term nitrate supplementation via beetroot juice improves treadmill walking performance and coronary hyperemic responses during plantar flexion exercise in patients with PAD. We hypothesized that 4 to 6 days of nitrate supplementation would improve oxygen supply to the heart and calf muscles of these patients leading to improved duration of treadmill walking and favorable effects on coronary hyperemic responses to plantar flexion. Secondary measures included blood pressure responses to exercise, plasma nitrate and nitrite concentrations, and methemoglobin (MetHb). We hypothesized that supplementation with nitrate via Nitrate-rich beetroot juice would increase plasma nitrite and attenuate the exaggerated rise in systemic blood pressure seen during leg exercise (treadmill and plantar flexion) in these patients.

## 2 Materials and methods

### 2.1 Study participants

These studies were completed in 6 patients enrolled between late 2015 and early 2017 (funded via an internal Penn State grant) and in 5 patients in 2019 and 2020 after additional funding was secured (R21); identical study design, nitrate supplementation procedures, and data collection methods were used for both cohorts. One patient in the first trial completed the screening visit but did not meet inclusion criteria. All 11 patients who met inclusion criteria completed the interventions and all follow-up study visits. The study interventions and all procedures for both trials were approved by the Institutional Review Board of the Penn State Health Milton S. Hershey Medical Center in agreement with the guidelines set forth by the Declaration of Helsinki. The study was also registered in 2015 on ClinicalTrial.gov (NCT02553733).

All participants provided written informed consent. Participants with PAD were recruited from the Penn State Heart and Vascular Institute Vascular Surgery Clinic. Patients with critical limb ischemia (rest pain, ulcers, or gangrene of the affected extremity) were excluded from the study and only PAD patients with their most recent ankle-brachial index (ABI) less than 0.9 were enrolled in this trial. All volunteers underwent extensive medical screening to assess eligibility. Exclusion criteria were: (1) allergy to beets, (2) diabetics with poor glycemic control (fasting glucose >126 mg/dL and/or HbA1C > 6.5% even with their medications), or any evidence of peripheral neuropathy; (3) history of unstable angina or myocardial infarction within 6 months of the study; (4) chronic kidney disease (eGFR <59 mL/min; Creatinine >2.0 mg/dL); (5) liver disease (ALT and AST 2x normal); (6) uncontrolled hypertension; (7) history of severe lung disease; and (8) history of bleeding or clotting disorders.

Demographic and clinical characteristics of the participants, which included 9 men and 2 women with documented PAD (Fontaine Stage IIa or IIb) are given in Table 1. All but one participant had a smoking history and 8 of the 11 participants indicated some habitual physical activity during the past year associated with yardwork (*n* = 3), walking (*n* = 5), and upper body exercise and/or occupational activity (*n* = 2). During the initial screening visit, all participants had elevated resting systolic blood pressure (>120 mmHg) or higher based on AHA’s current guidelines (29). Participants did not alter their medication routine during the beetroot interventions or prior to laboratory testing.

**Table 1:** Subject characteristics and medications.

		PAD		
		(*n* = 11)
Sex, n	Men/Women	9/2		
Age (years)		64.5 ± 8.0		
Height (cm)		174.1 ± 10.2		
Weight (kg)		82.0 ± 11.8		
BMI (kg/m^2^)		27.1 ± 3.9		
ABI	Left	0.73 ± 0.16		
	Right	0.74 ± 0.18		
SBP (mmHg)		139 ± 17		
DBP (mmHg)		81 ± 6		
MAP (mmHg)		100 ± 7		
Medications				
Plavix		3		
Aspirin		9		
Statins		9		
Antihypertensives		11		
Drugs labeled for PAD		4		

BMI, body mass index; ABI, ankle-brachial index; SBP, systolic blood pressure; DBP, diastolic blood pressure; MAP, mean arterial blood pressure. Data are expressed as mean ± SD.

### 2.2 Study protocol

**Figure 1:**
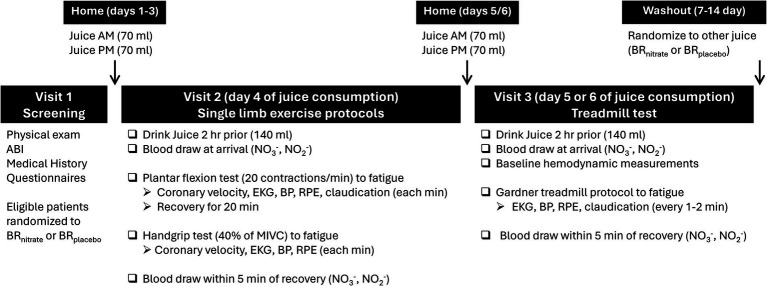
Timeline of experimental visits and juice consumption.

This study employed a double-blind, placebo-controlled, randomized, crossover design in which participants were compared to themselves. Participants were randomized using block size 2 and 4 to one of two treatment orders (active juice then placebo juice; placebo juice then active juice, with a 7–14 day washout; Figure 1). The two beetroot juices looked, smelled, and tasted the same and were supplied by James White Company in individually sealed bottles with identical color and labeling. The only difference between the juice treatments was the amount of nitrate, i.e., Beet-IT shot with 0.3 g of inorganic nitrate per 70 mL (BR_nitrate_) vs. placebo Beet-IT shot with 0.04 g of inorganic nitrate (BR_placebo_).

Participants were provided each juice treatment (i.e., enough for 7 days = 14 bottles) in a sequentially numbered, sealed, opaque lunch bag. All participants were given fluoride-free non-antibacterial toothpaste (Toms of Maine®, Kennebunk, Maine) to preserve nitrate reductase bacteria in oral cavity since these bacteria are needed to convert nitrate to nitrite (30). Research staff logged when each bag of juice was given to the participant, but the team was blinded to the treatment except one staff member with the randomization key. All visits were conducted in the morning hours in the Clinical Research Center (CRC) at the Penn State College of Medicine. The randomization code was disclosed after completing the initial trial (2015–2017) to facilitate preliminary power calculations for the subsequent R21 grant proposal. The code for the second, NIH-funded trial was disclosed after completing studies on 5 additional patients; a decision that was made necessary due to the COVID-19 pandemic-related shutdown of most human research protocols at Hershey Medical Center/CRC.

This study employed a total of 5 CRC visits in a randomized crossover design (Figure 1). The first visit consisted of a physical exam and screening measures to confirm study eligibility. Prior to each of the 4 subsequent CRC visits, participants consumed one 70 mL bottle of beetroot juice (either active or placebo) for 4–6 days each morning and evening at their home while taking their regularly prescribed medications. The range of 4 to 6 days allowed for continuous daily dosing over weekends when no clinical testing could be scheduled. Participants were asked to abstain from exercise, caffeine, and alcohol for 24 h and to undergo a water-only fast for at least 2 h prior to each study visit. Empty juice bottles were returned to a member of the research team after each arm of the study and recorded to ensure compliance.

### 2.3 Study measurements

During visits 2 and 4, approximately 2 h after the morning dose of juice consumption, participants performed isolated, graded, single-leg plantar flexion exercise in the supine position similar to that described previously (31). Participants had their most symptomatic foot strapped into a custom-made foot-pedal platform allowing rotation at the ankle. This foot pedal was connected via pushrods to a rotary device that lifted a weight pan during each plantar flexion followed by gravity-assisted return to the neutral position. Participants performed 20 plantar flexions/min guided by a metronome and feedback by a study investigator. The exercise was graded, starting at a workload of 2 kg, and was increased by 1 kg every minute up to 12 kg until participants were unable to maintain the cadence of the protocol or reached the maximum exercise duration of 14 min. The level of fatigue at the end of exercise was assessed using the Borg Rating of Perceived Exertion (RPE) scale (32).

Following a 20-min rest period, the participants completed isometric handgrip at 40% of their maximum force to volitional fatigue. For this test, patients were asked to squeeze a handgrip dynamometer as hard as possible to provide 3 voluntary contractions. The investigators then calculated 40% of the maximum voluntary contraction (MVC) for the handgrip bout to follow. Participants then performed the 40% MVC isometric handgrip exercise until their forearm muscles were totally fatigued. Participants were reminded to avoid the Valsalva maneuver.

Coronary blood flow velocity (CBV) of the left anterior descending (LAD) artery was estimated at rest and during both plantar flexion and handgrip using published methods to acquire CBV (33, 34). Briefly, CBV was obtained from the apical four-chamber view with a commercially available echocardiography system (Vivid 7, General Electric Healthcare). For this study, a variable frequency phased-array transducer (7S) was used employing color flow mapping and adjusted two-dimensional gain to enhance the blood flow signal from the LAD artery. Once the LAD signal was obtained, a 2.0-mm sample volume was placed over the color signal, and CBV was recorded at end-expiration. Despite not measuring LAD diameter due to limitations, previous studies have shown that CBV measurements via this method are comparable to those obtained by intracoronary Doppler guidewire and correlate with increases in coronary blood flow seen in angiograms (35). Heart rate and blood pressure were acquired continuously from a 3-lead EKG and a finger plethysmography cuff (Finapres) during all protocols.

Visits 3 and 5 included treadmill walking using the Gardner protocol, a validated protocol for use in PAD patients (36–38). During the treadmill exercise, a 12-lead EKG was placed to monitor cardiac rate/rhythm, while arm blood pressures were measured every 2 min by an automated auscultatory device (SunTech Tango). Patients reported when they first experienced leg discomfort (claudication onset time), when they could no longer continue walking (peak walking time), and rate of perceived exertion during the treadmill test (32, 39). Blood samples were drawn within 5 min after exercise for the measurement of plasma nitrate and nitrite.

### 2.4 Statistical analysis

Differences in resting hemodynamics, responses to exercise (CBV and blood pressures), perceived exertion, claudication onset time, and walking distances between the BR_placebo_ and BR_nitrate_ visits were assessed by paired t-tests. In cases where data did not meet the assumptions of normality, as indicated by the Shapiro–Wilk test and QQ plots, nonparametric statistical methods were employed. Specifically, the Wilcoxon matched-pairs signed rank test was utilized for paired data that did not follow a normal distribution. Between- and within-group changes in blood pressure responses, coronary hyperemic responses, and exercise duration were analyzed by fitting a mixed model rather than by repeated measures due to some missing values. Post-hoc comparisons between group factors were performed with Bonferroni’s method when an interaction effect was observed. The peak comparisons for changes from baseline were assessed via paired t-tests. Additionally, effect sizes were calculated using Cohen’s *d*, which quantifies the magnitude of the difference between two means in terms of standard deviations. Values of Cohen’s *d* are interpreted as small (0.2), medium (0.5), and large (0.8) effects, allowing for a standardized assessment of the practical significance of our findings. All statistical analyses were performed using Prism 7 (GraphPad Software, Inc., La Jolla, CA). All statistical tests were 1-sided and *p*-values <0.05 were considered significant, with appropriate adjustments made for multiple comparisons. All data are expressed as the mean ± standard deviation.

## 3 Results

There were no adverse events among the 11 participants including no nausea, vomiting, or diarrhea. There was also no clinically significant elevation in MetHb concentration after 4–7 days of beetroot juice consumption (0.7–1.8%, well within the normal range of 0–3%) (40).

**Figure 2:**
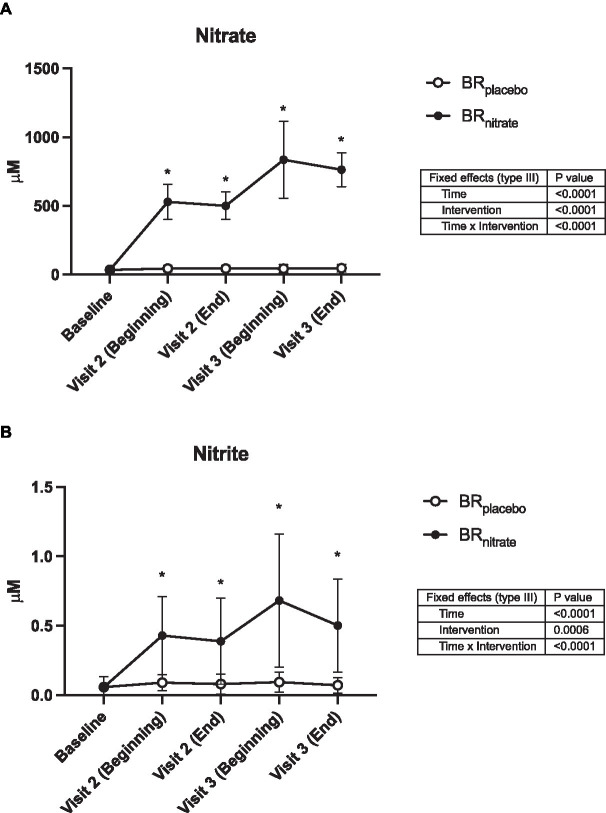
Plasma nitrate and nitrite concentrations. Data were collected at the baseline, the beginning, and the end of visit 2 (Day 4 of the juice consumption), the beginning and the end of visit 3 (Day 5 or 6 of the juice consumption). The plasma nitrate **(A)** and nitrite **(B)** concentrations were both significantly raised from baseline with nitrate-rich beetroot juice (black circle) and was significantly higher when compared to the nitrate-depleted placebo visit (white circle). BR_placebo_, nitrate-depleted beetroot juice; BR_nitrate_, nitrate-rich beetroot juice. Data are presented as means ± SD. *Significant difference from BR_placebo_, *p* < 0.05.

Plasma nitrate (NO_3_^−^) and nitrite (NO_2_^−^) concentrations each increased 14- and 7-fold, respectively, following nitrate supplementation (consumed at home) and remained high during laboratory testing, with negligible increases observed following placebo juice consumption (Figure 2).

### 3.1 Baseline measures

The baseline resting CBV for lab visit 2 of both treatments was not different (BR_placebo_ vs. BR_nitrate_; 20.2 ± 7.3 vs. 21.9 ± 4.9; *p* = 0.22 cm/s). Baseline resting DBP (BR_placebo_ vs. BR_nitrate_; 67 ± 10 vs. 62 ± 9 mmHg; *p* = 0.017) and MAP (BR_placebo_ vs. BR_nitrate_; 91 ± 9 vs. 85 ± 8 mmHg; *p* = 0.029) measured on the lab visit 2 were significantly lowered, while there was a trend for resting SBP (BR_placebo_ vs. BR_nitrate_; 140 ± 15 vs. 130 ± 15 mmHg; *p* = 0.06) to be reduced by dietary nitrate supplementation.

### 3.2 Plantar flexion and handgrip responses

**Figure 3:**
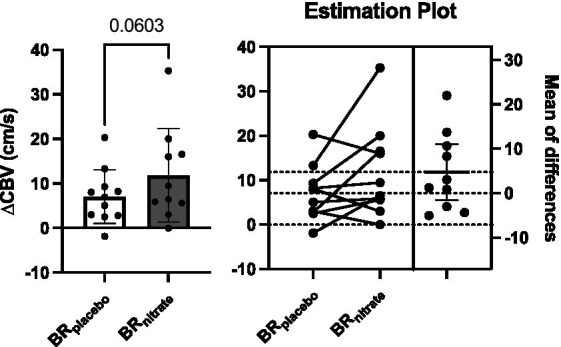
Effects of nitrate-rich beetroot juice on coronary blood flow velocity change from baseline to peak plantar flexion exercise. Left: Paired *t*-test was performed on delta CBV between BR_placebo_ and BR_nitrate_. Baseline CBV was not different between the intervention, but the change in CBV from baseline to peak plantar flexion exercise appears to be higher on the nitrate-rich beetroot juice visit compared to the placebo visit (*p* = 0.0603). Right: Individual delta CBV plot and the mean difference graphs. 7 of 10 PAD patients had greater increase in CBV at the peak workload of plantar flexion with nitrate-rich beet root juice. BR_nitrate_, nitrate-rich beetroot juice; BR_placebo_, nitrate-depleted beetroot juice; CBV, coronary blood flow velocity. Data are presented as means ± SD for the bar graph and as a mean ± 95% CI for the estimation plot.

Acute supplementation resulted in a trend for increased coronary hyperemic responses delta increase from baseline to plantar flexion exercise with BR_nitrate_ when compared to BR_placebo_ visit (BR_placebo_ vs. BR_nitrate_; 7.06 ± 6.01 vs. 11.83 ± 10.46 cm/s; *p* = 0.060; Cohen’s *d* = 0.56). The calf exercise time (BR_placebo_ vs. BR_nitrate_; 430.9 ± 160.6 vs. 425.5 ± 138.0 s; *p* = 0.41; Cohen’s *d* = 0.036; Figure 3), peak SBP (BR_placebo_ vs. BR_nitrate_; 163 ± 24 vs. 162 ± 30 mmHg; *p* = 0.48; Cohen’s *d* = 0.037) and peak DBP (BR_placebo_ vs. BR_nitrate_; 69 ± 12 vs. 69 ± 13 mmHg; *p* = 0.49; Cohen’s *d* = 0) were not different between the two visits.

No effects of BR_nitrate_ were observed during handgrip exercise on CBV (*p* = 0.33; Cohen’s *d* = 0.21) while handgrip exercise duration trended toward an increase (*p* = 0.06; Cohen’s *d* = 0. 516).

### 3.3 Treadmill responses

**Figure 4:**
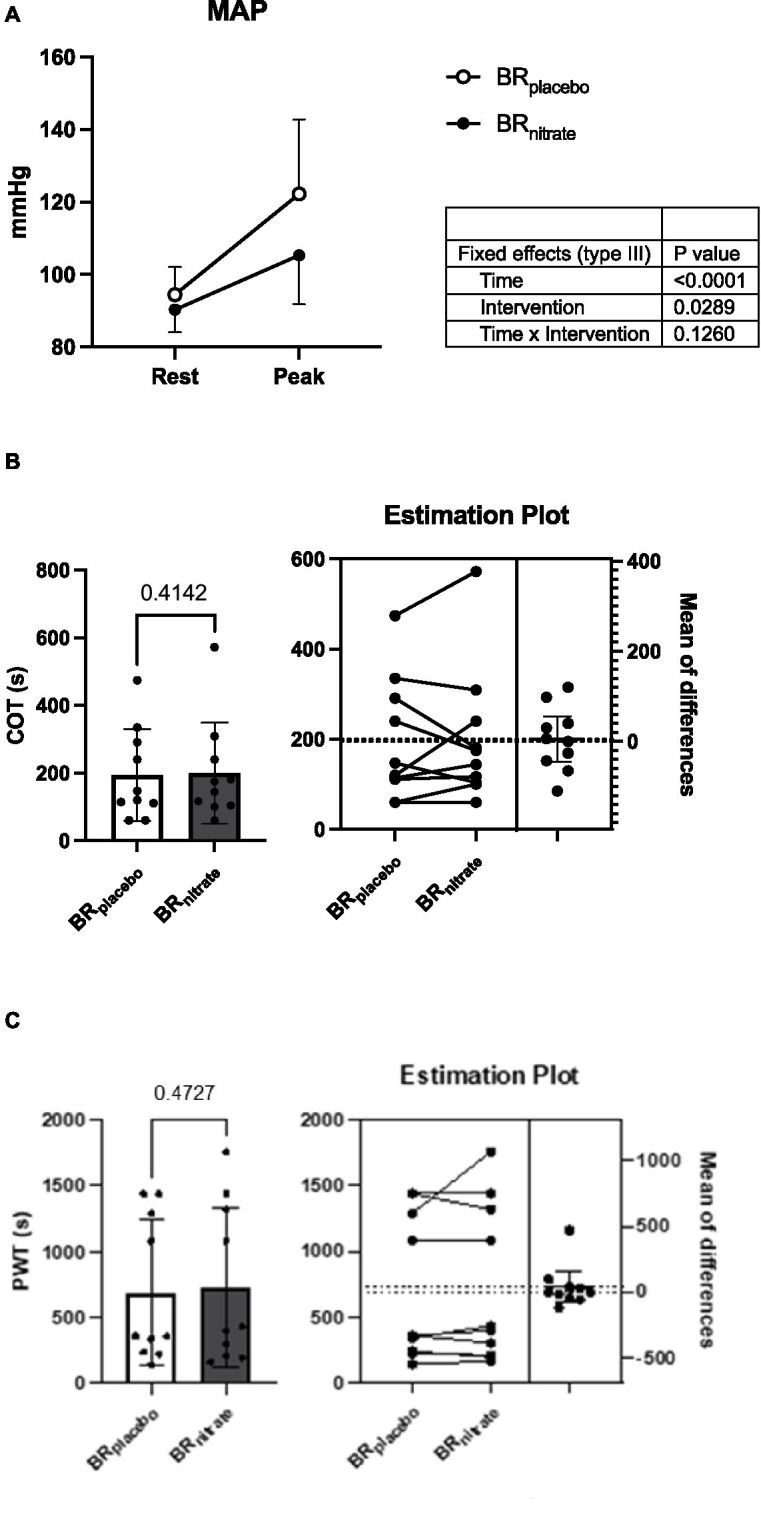
Effects of nitrate-rich beetroot juice on hemodynamics, claudication onset time, and peak walking time during treadmill walk. **(A)** Blood pressure response at rest and peak. **(B)** Claudication onset time on nitrate-depleted and -rich beetroot juice visits. **(C)** Peak walking time (PWT) on nitrate-depleted and -rich beetroot juice visits. Left: paired *t*-test was performed on COT and PWT between BR_placebo_ and BR_nitrate_. Both COT and PWT were not different between the interventions (*p* = 0.4142 and 0.4727, respectively). Right: individual plot shows 5 of 10 PAD patients had delayed COT and 4 of 10 PAD patients were able to walk longer during treadmill walk with BR_nitrate_; BR_nitrate_, nitrate-rich beetroot juice; BR_placebo_, nitrate-depleted beetroot juice; COT, claudication onset time; PWT, peak walking time. Data are presented as means ± SD for the bar graph and as a mean ± 95% CI for the estimation plot. *Significant difference from BR_placebo_, *p* < 0.05.

Peak walking time (PWT; BR_placebo_ vs. BR_nitrate_; 691.3 ± 547.7 vs. 730.5 ± 604.5 s; *p* = 0.473; Cohen’s *d* = 0.068) and claudication onset time (COT; BR_placebo_ vs. BR_nitrate_; 195.2 ± 136.1 vs. 200.2 ± 149.7 s; *p* = 0.41; Cohen’s *d* = 0.035) during treadmill testing were not different between the BR_placebo_ and BR_nitrate_ visits. The RPE was also not significantly different between the two visits (BR_placebo_ vs. BR_nitrate_; 15.3 ± 2.4 vs. 16.3 ± 2.5; *p* = 0.14; Cohen’s *d* = 0.41). However, the hemodynamic response during treadmill exercise was significantly improved, indicated by significantly lower DBP (BR_placebo_ vs. BR_nitrate_; 93 ± 19 vs. 80 ± 13 mmHg; *p* = 0.03), and MAP (BR_placebo_ vs. BR_nitrate_; 122 ± 21 vs. 105 ± 14 mmHg; *p* = 0.018) at the peak stages of the treadmill walking exercise during the BR_nitrate_ visit compared to BR_placebo_ visit (Figure 4). Additionally, there was a trend for SBP to be lower (BR_placebo_ vs. BR_nitrate_; 182 ± 27 vs. 156 ± 31 mmHg; *p* = 0.08) during the BR_nitrate_ visit.

No other variables were different between treatment conditions (*p* > 0.05). The pooled standard deviation of the change scores for each outcome is provided, permitting calculation of required sample sizes to detect meaningful changes in future studies (see Figures 3, 4).

## 4 Discussion

In this small randomized controlled clinical trial, we found that short-term consumption of nitrate-rich beetroot juice by older PAD patients did not improve their treadmill walking or isolated plantar flexion exercise duration relative to nitrate-depleted juice consumption, but did elicit favorable hemodynamic responses when myocardial demand was highest during peak treadmill walking. We also observed robust increases in plasma nitrite, a recognized source of NO (41) with short-term, twice daily nitrate-rich juice consumption in these patients. Overall, these preliminary findings suggest that inorganic nitrate supplementation in PAD patients is safe, well-tolerated, and may improve the coronary hyperemic and blood pressure responses when their calf muscles are most predisposed to ischemia.

### 4.1 Exercise performance responses

The lack of improvement in pain-free (claudication onset) or peak treadmill walk time in the present study was unexpected given previous individual reports showing improved walk test performance after acute (26, 27, 42) or chronic (25) inorganic nitrate supplementation in PAD patients. However, two additional supplementation studies, one using BR_nitrate_ acutely (28) (70 mL Beet-IT) and one that administered sodium nitrite tablets for 3 months (43) reported no significant improvements in walk distances relative to placebo. When examined collectively in a recent meta-analysis of studies investigating dietary nitrate and other upregulators of NO in PAD (44), it was reported that the improvements in maximum walk distance/duration did not achieve statistical significance. While there was an overall moderate improvement noted by these authors in claudication onset distance, when assessed across all 4 categories of NO-boosting supplements (i.e., NO donors including nitrate/nitrite, the NO modulators citrulline and/or arginine, NOS inducers, and antioxidants), the only supplement category that significantly improved claudication onset vs. placebo was antioxidants. The large variability in treadmill walk performance in PAD patients in general (20, 44) and in our small cohort in particular (Figure 3) coupled with the high antioxidant content of beetroot juice (45), are factors that likely diminished our ability to detect any potential improvements in walking performance in the present investigation. The large between-patient variability and the high content of antioxidants within both BR_nitrate_ and BR_placebo_ could also explain the lack of nitrate supplementation influence on exercise tolerance during our isolated plantar flexion testing.

### 4.2 Systemic hemodynamic (blood pressure) responses

Significant reductions in resting systolic and/or diastolic blood pressure have been reported following inorganic nitrate supplementation in healthy younger and older volunteers (46–49) hypertensive individuals (50, 51), and in cardiovascular disease populations, including PAD (25, 27, 28, 42). In the present study, we also observed a nitrate supplementation-dependent reduction in resting diastolic and mean BP at the start of the treadmill study visit (average 5 to 6 mmHg lower vs. placebo visit), with a trend for lower systolic BP as well. These results add to the literature regarding the blood pressure lowering potential of nitrate-rich beetroot juice in older adults with PAD.

It is well established that PAD patients have exaggerated blood pressure responses to exercise (1). This is most often observed during dynamic exercise involving the lower extremities (25, 52), although higher blood pressure responses have also been reported during moderate intensity rhythmic (11), but not isometric, forearm exercise (12). The mechanisms underlying exaggerated pressor responses in PAD are thought to involve increased, sympathetic nervous system activation to exercise via sensitized skeletal muscle afferents (53). Irrespective of the mechanisms, exaggerated blood pressure responses to dynamic exercise predispose PAD patients to an increased risk of adverse cardiovascular events (arrhythmias, myocardial infarction, and stroke) during and after exercise.

Surprisingly few studies have examined the impact of dietary nitrate supplementation on blood pressure responses during acute exercise in PAD patients. Kenjale et al. (42) were the first to report blood pressure lowering effects of a nitrate-rich supplement (9 mmol nitrate in 500 mL water; blood pressure medications withheld) in PAD patients, finding reductions in diastolic BP following acute nitrate consumption (relative to an orange juice consumption unblinded control visit) during submaximal (but not peak) treadmill walking. In the present study, which employed a more rigorous study design, we also observed lower exercise diastolic pressures, but only significantly during peak exertion. By contrast, in a recent study involving 18 PAD patients, acute consumption of nitrate-rich beetroot juice (6.5 mmol in 70 mL) had no impact on treadmill walking blood pressures up to peak effort (28). These heterogenous findings across studies with respect to exercise blood pressure point to the need for further investigations of acute and chronic blood pressure lowering effects of dietary nitrate supplementation in rigorously controlled trials of well-defined PAD patients.

### 4.3 Coronary responses

To our knowledge, the present study is the first to measure coronary vascular responses to exercise after nitrate supplementation in patients with PAD. While static handgrip increased CBV in these patients approximately 1.7–1.8-fold, consistent with our previous publication (53), the peak increases in CBV, and in systolic and diastolic blood pressures during the isometric handgrip test were not influenced by nitrate supplementation. This contrasts with the peak increase in CBV during plantar flexor exercise where, despite a lack of statistical significance, we observed a moderate effect size with increases evident in 7 of the 10 patients who had technically successful LAD coronary ultrasound imaging during both treatment conditions.

We have no direct insight into the mechanisms that may explain the apparent improvement in coronary hypermia during fatiguing calf (but not forearm) exercise in these patients. Myocardial oxygen demand and the magnitude of systemic nitrite reduction would, due to a larger active muscle mass and longer ischemic duration, presumably be greater during our plantar flexion test, possibly facilitating more NO-mediated coronary hyperemia and/or less NO-mediated central sympathoinhibition of the coronary vasculature (54). However, the direct effects of inorganic nitrite on the heart, including coronary artery dilation (55, 56) and improved diastolic function (57–59) as well as enhancement of collateral size and flow, decrease in afterload, and prevention or reversal of coronary artery vasoconstriction (60–62) could also contribute to the responses we observed. Evidence to date suggests that inorganic nitrite supplementation dilates predominantly large- and medium-sized coronary vessels (55, 56) with no effects on coronary microvessels (<100 micrometers) thereby minimizing any possibility of myocardial ischemia due to coronary steal (62).

### 4.4 Plasma nitrate and nitrite responses

Four days of BR_nitrate_ supplementation prior to study visit 1 resulted in an average 14-fold (*p* < 0.001) and 7-fold (*p* < 0.001) increase in plasma nitrate and nitrite, respectively, in these patients (Figure 2). These increases are consistent with acute and daily supplementation studies involving similar doses (i.e., ~10.5 mmol) in healthy older adults (24, 52, 63) and patient populations including PAD (64). An unexpected finding in the present investigation was the marked further increase in baseline (resting, fasted) nitrate and nitrite concentrations observed at the start of the second active (BR_nitrate_) study visit, an increase that apparently resulted from 1 to 2 additional days (average = 1.7 additional days) of nitrate consumption. Allen and colleagues did not observe increases in plasma nitrate or nitrite (beyond the first acute dose) in seven older PAD patients consuming nitrate-rich beetroot juice over a 3-month period (23). However, their patients consumed significantly less nitrate per week with lower frequency (i.e., 1 bottle/day on 3 days per week at 4.2 mmol per bottle) and participated in intermittent walking exercise after supplementation on those days, likely limiting plasma nitrate and nitrite accumulation. While the dose (two bottles/day) and frequency (4–6 consecutive days) of nitrate-rich juice consumption in our study is higher than most previous short-term supplementation studies in healthy and patient populations [typical doses have been 1 bottle/4–6 mmol per day over <7 days (65–67)] these results suggest that PAD patients have the capacity to augment circulating nitrate and nitrite if supplemented daily and if steps to avoid oral anti-bacterial practices are closely followed. The ability to increase plasma nitrite could be particularly important for PAD patients who appear to have a reduced ability (relative to healthy adults) to increase skeletal muscle nitrate and nitrite stores (68). Interestingly, chronic intake of dietary nitrate was recently shown to potentially alter the oral microbiome, promoting the growth of nitrate-reducing bacteria such as Neisseria and Rothia. These bacteria are crucial for the efficient reduction of nitrate to nitrite, thereby enhancing NO bioavailability. This alteration in the microbiome composition could be one of the mechanisms through which long-term nitrate supplementation exerts its beneficial effects on vascular health and exercise performance (69).

As shown in Figure 2, a trend was observed for the decrease in plasma nitrite during the day of the treadmill test, which was not evident during the day of the handgrip and plantar flexion testing. These visit 2 results, while not statistically significant, are consistent with earlier findings which reported that plasma nitrite declines significantly after intense cycling or running exercise, particularly after dietary nitrate supplementation in healthy adults (49, 70, 71) and after severe intensity exercise in hypoxia (70). Collectively, these plasma nitrate/nitrite findings provide further evidence that the metabolism of nitrate and nitrite in PAD patients is influenced by dietary nitrate supplementation and acute (large muscle mass) exercise.

### 4.5 Experimental strengths and limitations

In the present study, we used a double-blind, placebo-controlled, randomized crossover study design with rigorously screened patients who consumed a verified source and sufficient daily dose of nitrate to elicit physiological effects. Our participation also followed strict pre-visit instructions to maximize oral conversion of nitrate-to-nitrite including the avoidance of gum and mouthwash and daily use of a non-antibacterial toothpaste that we provided to the patients. While we did not strictly control the participants’ diet, they did record their food in a daily log during each intervention and they were instructed to consume only water for at least 8 h prior to each study visit. These patients consumed the juice at home and continued taking their regular medications. The robust increases in plasma nitrate and nitrite confirm patient compliance with these study instructions (also confirmed by daily juice consumption log sheets and returned empty bottles) and indicate significant physiological conversion of nitrate-to-nitrite. An advantage of using the crossover study design, which has to our knowledge only been employed in two of the previous dietary nitrate PAD trials (27, 42), is that participants served as their own controls.

Despite the experimental rigor and novel findings of this study, there are several limitations to acknowledge. First is the small sample size and the combining of data from two sequential trials separated by about 2 years. As indicated above in the Methods, both trials were identical in terms of the juice intervention, measurement procedures, and study execution including involvement of most of the same investigative team members (including the same ultrasonographer) across both trials. While limited by several logistical factors, most notably the disruption of participant recruitment associated with the COVID-19 pandemic and the discontinuation of clinical trials at our institution at that time, the data collected in this sample of patients provides effect size and standard deviation change scores that can be used to sufficiently power future interventional trials with these methods. It is also important to note that our samples had only, 2 female participants and was limited to White/Caucasian participants. Future dietary nitrate supplementation trials in this patient population should include more ethnic diversity and more women as they exhibit greater functional limitations than men with a similar ABI (72) and who may also have greater nitrate-reducing bacteria in their saliva compared to men (73).

The use of the transthoracic Doppler technique to measure coronary hyperemic responses also has some limitations. First, it is motion sensitive and requires the torso to be stationary, thus limiting its use to isolated, single limb exercise. This measurement, like all ultrasound-based methods, requires considerable technician experience. Technical success of ultrasound-derived LAD velocity measurements in our laboratory is 80–90% with good test–retest reliability (ICC =0.82–0.90). We should also note that this technique provides measures of blood flow velocity rather than absolute blood flow, and in only the LAD coronary vessel. However, this technique has been validated against invasive measurements (74). Additionally, changes in coronary velocity are 10 times greater than changes in diameter of the LAD during physiological stressors (75).

Lastly, there were 2 patients with missing blood pressure data during the final stage of the plantar flexion test for one visit due to excessive upper body movement. Thus, the decision was made to use the next to last stage blood pressure measurement to represent the “peak” plantar flexion blood pressure. Both visits were BR_nitrate_ visits.

### 4.6 Clinical translational perspectives

Unlike organic nitrate administration (nitroglycerin, nitroprusside), dietary supplementation with nitrate-rich juice/foods or direct oral nitrite administration facilitates the targeted delivery of NO to ischemic tissues (43) and does not lead to tachyphylaxis/nitrate tolerance with repeated dosing (51). Increasing bioavailable NO with plant-based beverages/foods is also associated with fewer side effects than organic nitrates (or nitrite salts), including acute hypotension; this is likely due to the more gradual process of nitrite creation (76) and reduction to NO at selected locations and times of increased tissue oxygen demand. These therapeutic advantages, along with the fact that nitrite acts in a manner that is unlikely to induce coronary steal, supports further investigation into beetroot juice as a potential adjunctive treatment for the large and growing number of older adults with this disabling disease.

The double-blinded use of nitrate-depleted (true placebo) beetroot juice has been the gold standard for determining the physiological and exercise performance effects of dietary nitrate supplementation in healthy and patient groups (44, 46, 75, 77, 78). However, the high antioxidant content of beetroot juice, which has itself been found to improve walking tolerance in PAD (44, 79), could obscure the ability to detect the ergogenic potential of beetroot juice when only nitrate-depleted beetroot juice is used as the placebo control. It may be important, therefore, for future studies examining the effects of beetroot juice on exercise tolerance in PAD and other patient groups with high systemic oxidative stress, to include a third study visit/condition (e.g., a nitrate-rich, low antioxidant beverage) as a non-beetroot juice control.

## 5 Summary and conclusion

In this small study of PAD patients, short-term nitrate supplementation with beetroot juice did not improve leg exercise tolerance, but robustly increased plasma nitrite and resulted in lower diastolic and mean arterial pressure during strenuous uphill treadmill walking. We also report, for the first time in any patient population, evidence for an improved coronary hyperemic response to exercise after nitrate supplementation. Overall, these preliminary findings suggest that inorganic nitrate supplementation in PAD patients is safe, well-tolerated, and may improve the coronary hyperemic and blood pressure responses when their calf muscles are most predisposed to ischemia.

## Data availability statement

The raw data supporting the conclusions of this article will be made available by the authors, without undue reservation.

## Ethics statement

The studies involving humans were approved by the Penn State Human Research Protection Program. The studies were conducted in accordance with the local legislation and institutional requirements. The participants provided their written informed consent to participate in this study.

## Author contributions

DK: Conceptualization, Data curation, Formal analysis, Investigation, Methodology, Project administration, Validation, Visualization, Writing – original draft, Writing – review & editing. ZG: Data curation, Formal analysis, Investigation, Methodology, Writing – original draft, Writing – review & editing. JL: Data curation, Formal analysis, Software, Writing – original draft, Writing – review & editing. KB: Data curation, Formal analysis, Software, Writing – original draft, Writing – review & editing. AM: Data curation, Formal analysis, Supervision, Writing – original draft, Writing – review & editing. DK-S: Funding acquisition, Methodology, Resources, Supervision, Writing – original draft, Writing – review & editing. SB: Methodology, Resources, Writing – original draft, Writing – review & editing. UL: Conceptualization, Funding acquisition, Methodology, Resources, Supervision, Writing – original draft, Writing – review & editing. AG: Conceptualization, Methodology, Resources, Supervision, Writing – original draft, Writing – review & editing. MM: Conceptualization, Data curation, Funding acquisition, Investigation, Methodology, Resources, Supervision, Writing – original draft, Writing – review & editing. DP: Conceptualization, Funding acquisition, Methodology, Resources, Supervision, Writing – original draft, Writing – review & editing.
